# *Rosmarinus officinalis* L. hexane extract: phytochemical analysis, nanoencapsulation, and in silico, in vitro*,* and in vivo anti-photoaging potential evaluation

**DOI:** 10.1038/s41598-022-16592-7

**Published:** 2022-07-30

**Authors:** Nehal Ibrahim, Haidy Abbas, Nesrine S. El-Sayed, Heba A. Gad

**Affiliations:** 1 Pharmacognosy Department, Faculty of Pharmacy, Ain Shams University, Cairo, 11566 Egypt; 2 Pharmaceutics Department, Faculty of Pharmacy, Damanhour University, Damanhour, Egypt; 3 Pharmacology and Toxicology Department, Faculty of Pharmacy, Cairo University, Cairo, Egypt; 4 Pharmaceutics and Industrial Pharmacy Department, Faculty of Pharmacy, Ain Shams University, Cairo, 11566 Egypt; 5 Department of Pharmaceutical Sciences, Pharmacy Program, Batterjee Medical College, Jeddah, Saudi Arabia

**Keywords:** Skin diseases, Chemistry, Nanoscience and technology

## Abstract

A shift towards natural anti-aging ingredients has spurred the research to valorize traditionally used plants. In this context, *Rosmarinus officinalis* L. was evaluated for its photoprotective, antioxidant, anti-inflammatory, and anti-wrinkling properties. GC/MS and LC-ESI-HRMS based phytochemical profiling of rosemary leaves hexane extract resulted in the identification of 47 and 31 compounds, respectively and revealed rich content in triterpenoids, monoterpenoids and phenolic diterpenes. In vitro assays confirmed the antioxidant, anti-aging, and wound healing potential of rosemary extract along with a good safety profile, encouraging further development. A systematic molecular modelling study was conducted to elucidate the mechanistic background of rosemary anti-aging properties through the inhibitory effects of its major constituents against key anti-aging targets viz. elastase, collagenase, and hyaluronidase. Development of rosemary extract lipid nanocapsules-based mucoadhesive gels was performed to improve skin contact, permeation, and bioavailability prior to in vivo testing. The developed formulae demonstrated small particle size (56.55–66.13 nm), homogenous distribution (PDI of 0.207–0.249), and negatively charged Zeta potential (− 13.4 to − 15.6). In UVB-irradiated rat model, topical rosemary hexane extract-loaded lipid nanocapsules-based gel provided photoprotection, restored the antioxidant biochemical state, improved epidermal and dermal histological features, and decreased the level of inflammatory and wrinkling markers. The use of rosemary hexane extract in anti-aging and photoprotective cosmeceuticals represents a safe, efficient, and cost-effective approach.

## Introduction

Skin aging is a multifactorial physiological and pathophysiological phenomenon affected by genetic, hormonal, metabolic, and environmental factors. Since skin is constantly exposed to the environment, combatting skin damage due to the solar UV radiation has been gaining considerable interest especially with the increase in human life expectancy. Repeated exposure to solar UV radiation results in cleavage and disorganization of skin connective tissue with subsequent sagging and appearance of crevices and wrinkles. In addition, skin aging is accompanied with impaired wound healing and pigmentation changes^1^.

Due to consumers’ growing concerns about synthetic products and their possible detrimental health effects, there has been a shift during the last decades towards the implementation of natural bioactive, functional ingredients or additives in cosmetics and personal care products^2–5^. Such preference for natural cosmeceuticals is maximized when they are derived from edible sources and culinary herbs^4,6^ and was reflected on the European market of natural cosmetics which was valued at 4 billion dollars in 2015 and further expanded globally between 2015 and 2019, with up to 11% annual growth^3,4,7^. Many of such natural ingredients exhibit antioxidant, anti-inflammatory, and antimicrobial properties. Rosemary (*Rosmarinus officinalis* L.) is a Lamiaceae shrub widely distributed in the Mediterranean region where its use dates back to the pharaonic era^8^. The ancient Egyptians used rosemary together with chamomile, myrrh, and thyme to protect skin against desert heat^9^. In addition, *R. officinalis* has long history of use in phytotherapeutical traditions as antispasmodic, diuretic, antirheumatic, and antiepileptic, and showed effectiveness in treating respiratory problems and skin infections and enhancing wound healing^8^. Moreover, during the World War II, rosemary leaves were burnt to disinfect hospitals^10^.

Recently, a plethora of biological activities have been reported to rosemary essential oils and to the polar phenolics extracts (prepared by methanol, ethanol, and acetone), including antioxidant, anti-inflammatory, hypoglycemic, antimicrobial, hepato-, nephro-, and neuroprotective, memory enhancing, wound healing, and anti-wrinkle properties, validating many of its traditional uses^11–19^. All these activities were attributed to rosemary’s complex repertoire of volatile constituents, phenolic diterpenes, e.g., carnosol and carnosic acid; and polyphenols, e.g., rosmarinic acid^11,13,19–22^. Nevertheless, quite surprisingly less interest has been paid to rosemary hexane extracts compared with the large body of literature available on rosemary essential oil and alcoholic extracts^10–12,20,23,24^. A lot of research has been conducted and published on rosemary essential oil or alcoholic extracts while the hexane extracts with their uniquely different composition remain much less explored. Previous studies have documented the superior antioxidant activity and higher carnosic acid content of rosemary hexane extract compared with rosemary phenolics extracts prepared with methanol, ethanol, and acetone^23,24^. In addition, hexane efficiently extracts the aroma compounds, e.g., monoterpenes, sesquiterpenes, phenylpropenes, and their oxygenated derivatives which may exhibit antioxidant and antiaging properties^25–27^ and further improve consumer acceptability. Furthermore, unlike polar solvents, hexane can extract triterpenes which exhibit anti-inflammatory properties^28^, tocopherols, and saturated and unsaturated fatty acids which exhibit anti-inflammatory, antioxidant, antiaging, and permeation enhancing effects^29,30^. However, *R. officinalis* hexane extract is regarded as a byproduct during the agri-food processing of rosemary for the production of decolorized polyphenol-rich extracts for commercial exploitation as natural antioxidants and antimicrobials in food preservation. This results in the generation of significant volumes of hexane extract wastes^31^. With the initiative of circular economy, such wastes can be valuable sources of phytochemicals needed in different industrial and medicinal applications, which necessitates their valorization to render the process more sustainable and maximize economic and environmental benefits^5,31,32^.

Lipid nanocapsules (LNC) are newly evolved lipid-based nanocarriers formed of an oily core of medium chain triglycerides that is enclosed within a shell of PEGylated surfactant and lipoid. The oily core of LNC increases their ability to encapsulate lipophilic compounds. In addition, their small particle size (20-100 nm) grants high skin permeation upon dermal application. Other advantages include safety, biocompatibility, ease of preparation, and good stability^33,34^.

The present work is undertaken to unveil the compositional profile, the in vitro anti-aging, antioxidant, and wound healing potentials as well as the cytotoxic effect of *Rosmarinus officinalis* L. hexane extracts, and to improve its skin permeation and bioavailability through inclusion into lipid nanocapsules-based gel. In addition, the cosmeceutical potential of formulated and unformulated *R. officinalis* hexane extract was evaluated in vivo with regard to its UV-protection capacity and antioxidant, anti-inflammatory, and anti-wrinkling properties. Moreover, a systematic molecular modelling study was conducted to elucidate the mechanistic background of the anti-aging properties of rosemary hexane extract.

## Results and discussion

### Chemical profiling of *R. officinalis* hexane extract

#### GC-MS analysis

GC-MS analysis was implemented to assess the metabolite composition of rosemary hexane extract (RHE) revealing the presence of monoterpenes, their oxygenated derivatives, sesquiterpenes, long chain alkanes, and triterpenoids and resulting in the identification of 47 components representing 99.29% of detected peaks (Table 1). Triterpenes (45.2%) and hydrocarbons (41.8%) dominated the extract with α-amyrin, dotriacontane, β-amyrin, and triacontane as major components. Volatile constituents of rosemary aroma were mainly represented by monoterpenes and their oxygenated derivatives detected at 3% and 8.4%, respectively. The chief monoterpene was α-pinene, while the major oxygenated monoterpenes included the ketones verbenone and camphor, besides 1,8-cineole and borneol. This is congruent with previous reports of major terpenes of rosemary essential oil^11^. Lipophilic hexane extracts of aromatic plants exhibit aroma profile that closely mimics the characteristic odour of fresh plant. On the contrary, the aroma quality of volatiles prepared by distillation may differ from the fresh raw material because of the high temperatures used in distillation procedures^36,37^.

**Table 1:** Compositional profile of *R. officinalis* hexane extract as analysed by GC-MS.

No	RT (min)	RI exp^a^	RI lit^b^	Metabolite^c^	Relative percentile
1	7.07	914	914	*α*-Pinene	1.73
2	7.52	929	929	Camphene	0.46
3	7.7	936	937	2,4(10)-Thujadiene	0.1
4	8.39	960	960	*β*-Pinene	0.13
5	9.45	998	998	*δ* 3-carene	0.08
6	9.93	1013	1013	*p*-Cymene	0.07
7	10.05	1017	1017	Limonene	0.4
8	10.11	1019	1019	Eucalyptol	1.63
9	12.34	1089	1089	Linalool	0.45
10	13.73	1133	1133	Camphor	2.07
11	14.32	1152	1152	Pinocarvone	0.12
12	14.42	1155	1155	Borneol	1.07
13	14.68	1163	1162	Isopinocamphone	0.17
14	15.22	1180	1180	*α*-Terpineol	0.21
15	15.59	1192	1196	Isoborneol	0.09
16	15.77	1198	1198	Verbenone	2.08
17	16.78	1233	1234	*cis*-Myrtanol	0.21
18	18.02	1276	1276	Bornyl acetate	0.31
19	21.8	1408	1408	Caryophyllene	0.2
20	42.19	2368	2300	Tricosane	0.21
21	43.84	2467	2400	Tetracosane	0.27
22	44.78	2528	2525	Diisooctyl phthalate	0.19
23	45.42	2569	2500	Pentacosane	0.43
24	46.93	2666	2600	Hexacosane	0.8
25	48.39	2760	2700	Heptacosane	0.65
26	49.83	2853	2800	Octacosane	3.07
27	51.18	2939	2865	2-methyloctacosane	0.9
28	52	2992	2965	2-Methylnonacosane	0.35
29	52.53	3026	3000	Triacontane	6.73
30	53.29	3075	3112	*α*-Tocopherol	0.34
31	53.49	3087	3015	3,7-dimethyl-nonacosane	0.54
32	53.83	3110	3100	Hentriacontane	1.34
33	54.58	3157	3120	n-Octacosanol	0.21
34	54.73	3167	3100	Hentriacontane	1.26
35	55.36	3208	3200	Dotriacontane	16.21
36	56.34	3270	3225	16-Methyldotriacontane	0.36
37	56.56	3285	3235	12-Methyldotriacontane	1.7
38	56.69	3294	3337	*β*-Amyrin	10.4
39	56.99	3313	3300	Tritriacontane	1.11
40	57.1	3319	3337	*β*-Amyrin	3.95
41	57.57	3350	3376	*α*-Amyrin	20.64
42	58	3377	3376	*α*-Amyrin	3.79
43	58.19	3389	3338	15-Methyltritriacontane	0.71
44	58.98	3440	3400	Tetratriacontane	5.11
45	59.35	3464	3384	Lupenone	0.67
46	62.4	3660	3525	Lupeol acetate	4.91
47	62.62	3674	3629	Betulinaldehyde	0.86
% Total Identified					99.29
% Monoterpenes					2.97
% Oxygenated monoterpenes					8.41
% Sesquiterpenes					0.2
% Hydrocarbons					41.75
% Triterpenoids					45.22
Others					0.74

^a^Retention index calculated experimentally on Rtx-5MS column relative to C8-C28 n-alkanes series.

^b^Corresponding Kovats retention index from literature and spectral databases.

^c^Identification based on comparing retention indices (RI) and mass spectral data (MS) with those found in NIST Mass Spectral Library (2011), Wiley Registry of Mass Spectral Data (8^th^ edition) and reported in literature.

Triterpenoids were dominated by α-amyrin (24.4%), β-amyrin (14.4%), and lupeol acetate (4.9%). α-Amyrin, a pentacyclic triterpene alcohol previously identified as minor component in *R. officinalis* ethanol extract^38^, showed anti-inflammatory, anti-allergic, antihyperlipidemic, and anti-ulcer activities^39–42^. In vitro, this triterpenoid stimulated human keratinocytes proliferation^43^. Lupeol acetate demonstrated anti-inflammatory, antinociceptive, and anti-arthritic activities^44,45^.

#### LC-ESI-HRMS analysis

*Rosmarinus officinalis* leaves hexane extract was profiled for its phytochemical composition using LC-ESI-HRMS. The compounds were tentatively identified by comparing their corresponding retention times and HRMS data with those previously reported in literature and online databases. The LC-ESI-HRMS analysis resulted in the tentative identification of 31 metabolites from different classes. Phenolic diterpenes (e.g., rosmanol, carnosol, carnosic acid, and rosmadial) represent the most abundant class detected in RHE with 14 identified compounds in accordance with previous studies^46,47^, followed by triterpenoids (e.g., betulinic, oleanolic and ursolic acids) represented here by 9 compounds. The identified compounds and their chromatographic and HRMS data characteristics are depicted in Table 2.

**Table 2:** Phytochemical composition of *R. officinalis* hexane extract as analysed by LC-ESI-HRMS in negative ion mode.

No	RT (min)	Annotation	Molecular formula	Exp. [M-H]^−^ *m/z*	Exact mass	MS/MS	Error (ppm)	Class	Refs.
1	19.234	Coniferyl alcohol	C_10_H_12_O_3_	179.0712	180.0786	nd	0.49	Phenylpropanoid	^52^
2	26.094	Podolide	C_19_H_22_O_5_	329.1405	330.1467	nd	− 4.28	Norditerpene	^53^
3	29.12	Rosmanol	C_20_H_26_O_5_	345.1717, 691.3501 [2 M-H]^−^	346.178	283, 301	− 1.76	Phenolic diterpene	^46,47^
4	30.331	(Epi)(iso)rosmanol I	C_20_H_26_O_5_	345.1718, 691.3507 [2 M-H]^−^	346.178	283, 301	− 3.09	Phenolic diterpene	^46,49^
5	31.441	(Epi)(iso)rosmanol II	C_20_H_26_O_5_	345.1719	346.178	283, 301	− 3.14	Phenolic diterpene	^46^
6	34.568	Lariciresinol	C_20_H_24_O_6_	359.1509	360.1573	nd	− 2.48	lignan	^54^
7	35.072	Unidentified	C_19_H_22_O_4_	313.1452	314.1518	nd	− 2.15		
8	36.485	Rosmadial	C_20_H_24_O_5_	343.1563, 687.3191 [2 M-H]^−^	344.1624	299, 315, 313	− 3.34	Phenolic diterpene	^47^
9	37.494	(Epi)rosmanol methyl ether	C_21_H_28_O_5_	359.1877	360.1937	344, 315, 329	− 3.36	Phenolic diterpene	^46,55^
10	38.099	Carnosol	C_20_H_26_O_4_	329.1772, 659.3604 [2 M-H]^−^	330.1831	285	− 3.95	Phenolic diterpene	^56,57^
11	39.713	Rosmadial isomer	C_20_H_24_O_5_	343.1565	344.1624	299, 315	− 3.71	Phenolic diterpene	^47^
12	40.621	Unidentified	C_19_H_34_O_4_	325.2395	326.2457	nd	− 2.94		
13	40.722	Rosmaridiphenol	C_20_H_28_O_3_	315.1977, 631.4020 [2 M-H]^−^	316.2038	285, 135	− 3.58	Phenolic diterpene	^46^
14	40.823	Carnosic acid	C_20_H_28_O_4_	331.1928, 663.3922 [2 M-H]^−^	332.1988	244	− 3.91	Phenolic diterpene	^46,48,57^
15	40.924	Rosmaridiphenol isomer I	C_20_H_28_O_3_	315.1980, 631.4024 [2 M-H]^−^	316.2038	nd	− 4.43	Phenolic diterpene	^46^
16	41.025	Rosmaridiphenol isomer II	C_20_H_28_O_3_	315.1977	316.2038	nd	− 3.46	Phenolic diterpene	^46^
17	42.03	Unidentified	C_23_H_32_O_3_	355.2291	356.2351	nd	− 3.21		
18	42.74	Carnosol isomer	C_20_H_26_O_4_	329.1770, 659.3601 [2 M-H]^−^	330.1831	nd	− 3.37	Phenolic diterpene	^46,57^
19	43.648	Asiatic acid	C_30_H_48_O_5_	487.3444, 975.6934 [2 M-H]^−^	488.3502	nd	− 2.95	Triterpenoid	^58^
20	45.06	12-O-methylcarnosic acid	C_21_H_30_O_4_	345.2080	346.2144	301	− 2.85	Phenolic diterpene	^47^
21	45.665	Carnosic acid isomer	C_20_H_28_O_4_	331.1916	332.1988	nd	− 0.38	Phenolic diterpene	^46,57^
22	47.3	Asiatic acid isomer	C_30_H_48_O_5_	487.3438	488.3502	nd	− 2.29	Triterpenoid	
23	47.683	betulinic acid	C_30_H_48_O_3_	455.3543	456.3603	455, 411	− 2.7	Triterpenoid	^47,59^
24	48.288	Lanopalmitic acid	C_16_H_32_O_3_	271.2283	272.2351	nd	− 1.54	Hydroxy fatty acid	^54^
25	48.49	Unidentified triterpenoid	C_30_H_46_O_3_	453.3378	454.3447	nd	− 0.73	Triterpenoid	
26	48.995	Oleanolic acid	C_30_H_48_O_3_	455.3537	456.3603	407	− 1.25	Triterpenoid	^47,59^
27	50.810	Ursolic acid	C_30_H_48_O_3_	455.3540	456.3603	nd	− 1.26	Triterpenoid	^47,59^
28	51.618	Unidentified triterpenoid	C_30_H_44_O_3_	451.3230, 903.6532 [2 M-H]^−^	452.329	nd	− 3.07	Triterpenoid	
29	52.626	Unidentified triterpenoid	C_30_H_46_O_3_	453.3390, 907.6833 [2 M-H]^−^	454.3447	nd	− 2.84	Triterpenoid	
30	59.89	Augustic acid	C_30_H_48_O_4_	471.3498	472.3553	nd	− 2.79	Triterpenoid	^55^
31	72.298	Pyro-pheophytin-b	C_53_H_70_N_4_O_4_	825.5338	826.5397	nd	− 7.15	Chlorophyll derivative	^60^

Compounds 3, 4, 5, and 20 exhibited quasimolecular ions [M-H]^−^ at m/z 345. Compounds 3, 4, and 5 were tentatively identified as rosmanol, (epi)(iso)rosmanol I, and (epi)(iso)rosmanol II based on the accurate masses of the observed deprotonated quasimolecular ions (m/z 345.171) and deprotonated dimers [2 M-H]^−^ (m/z 691.3501). Additionally, fragment ions were detected at m/z 301, and 283 which correspond to the loss of CO_2_ and sequential loss of CO_2_ and water, respectively, in line with previously reported data for these isomeric compounds^46,48^. Compound 20 was identified as 12-O-methylcarnosic acid. Although it showed the same nominal mass with [M-H]^−^ at m/z 345, it presented a different accurate mass of 345.2080 and eluted later in the chromatographic separation which is previously reported for 12-O-methylcarnosic acid^48,49^. Fragment ion at m/z 301 indicated the loss of CO_2_ from the carboxylic acid group^48^.

Rosmadial (compound 8) and its isomeric form (compound 11) showed quasimolecular ions at m/z 343.1563 and 343.1565, respectively. A unique fragmentation pattern was recorded for these compounds; loss of ethylene, CO_2_ and HCHO can account for the product ions observed at m/z 315, 299 and 313, respectively, as formerly described^46,48,49^. Compound 9 was identified as (epi)rosmanol methyl ether based on its [M-H]^−^ accurate mass of 359.1877 and its fragment ions at m/z 344, 329 and 315 attributed to loss of methyl, HCHO and CO_2_^50^. Carnosol (compound 10) as well as its isomer (compound 18) were identified based on the accurate masses of their [M-H]^−^ and [2 M-H]^−^ and the characteristic fragment ion at m/z 285 ascribed to the loss of CO_2_. Rosmaridiphenol (compound 13) was identified based on the accurate mass of its [M-H]^−^ observed at m/z 315.1977 and its fragment ion at m/z 285, which is consistent with previous reports^46,51^.

Carnosic acid (compound 14) and its isomer (compound 21) were identified by their corresponding deprotonated quasimolecular ions observed at m/z 331.1928 and 331.1916, respectively. Carnosic acid produced a fragment ion at m/z 244 which can be attributed to the loss of CO_2_ + CH_3_CH_2_CH_2_, as previously reported for this highly antioxidant phenolic diterpene^48,49^.

### In vitro antioxidant capacity

Oxidative stress is a key element in the aging process as well as in the aetiology of various chronic disorders through its inflammatory and degenerative consequences. Topical application of antioxidants can restore the balance between antioxidation and oxidation processes, prevent molecular damage, and maintain skin homeostasis. In this regard, plant extracts provide a wide range of antioxidant molecules ranging from phenolic acids, flavonoids, and tannins to carotenoids, tocopherols, and terpenoids. It is noteworthy that the extraction solvent has a strong impact on the antioxidant activity of a plant extract. Although polar solvents, e.g., methanol and ethanol, are best suited for extraction of polyphenols and better reflect the antioxidant potential of a plant^61^, the n-hexane extract of certain plants, including rosemary, demonstrated better antioxidant activity than their polar extracts based on in vitro chemical antioxidants tests^23,24,62^.

The antioxidant activity was assessed using DPPH and ABTS free radicals scavenging assays as well as FRAP assay. RHE was able to scavenge DPPH radical (IC_50_ of 221.6 ± 11.8 µg/mL), but it was less active than Trolox (IC_50_ of 6.1 ± 0.2 µg/mL) (Table 3). The DPPH scavenging activity of RHE is comparable to *Ipomoea cairica* and *Bauhinia purpurea* hexane extracts^63^. However, it is much less than the DPPH scavenging capacity of rosemary essential oil (IC_50_ of 77.6 µL/mL)^64^. Meanwhile, it is worth mentioning that this antioxidant activity is comparable and even superior to published antioxidant properties of phenolics rich extracts^65,66^.

**Table 3:** Antioxidant activity of *R. officinalis* hexane extract.

Sample	DPPH (IC_50_, µg/mL)	ABTS (mM TE/g extract)	FRAP (mM TE/g extract)
RHE	221.6 ± 11.8	310.54 ± 12.32	394.69 ± 17.28
Trolox	6.11 ± 0.2	–	–

The free radical scavenging capacity of RHE was also evaluated using the ABTS decolorization assay. This is based on the capacity of antioxidant compounds to scavenge the radical cation ABTS^•+^ relative to the standard antioxidant Trolox. RHE demonstrated a Trolox equivalent antioxidant capacity of 310.5 ± 12.3 mM TE/g dry extract (Table 3), well above the previously reported antioxidant capacity of *Vitex agnus-castus* leaves and fruits hexane extract^67^.

The FRAP assay is used to evaluate the reducing power of compounds and depends on the ability of antioxidants in plant extracts to reduce the colourless Fe^3+^‐TPTZ complex to the blue coloured Fe^2+^‐TPTZ complex. In FRAP assay, RHE demonstrated a Trolox equivalent antioxidant capacity of 394.7 ± 17.3 mM TE/g dry extract (Table 3). The different antioxidant results obtained from the three assays may reflect differences in the capacity of compounds in the extract to quench DPPH and ABTS free radicals and to reduce ferric ion in vitro. Among the methods used, ABTS and FRAP assays are the most correlated, a finding that was formerly reported^68,69^. It should be noted that the antioxidant assays are preferably performed in the context of the whole organism to obtain more reliable information^70^, which is complemented in the present work by in vivo biochemical analysis.

### In vitro anti-aging potential

Collagen, elastin, and hyaluronic acid represent the major structural components of the dermal extracellular matrix (ECM). Making up 80% of the skin dry weight, collagen is responsible for the skin tensile strength^71^. Brittle when dry but flexible and elastic when moist, elastin fibres maintain skin elasticity^72^. In addition, the mucopolysaccharide hyaluronic acid supports skin viscoelasticity, smoothness, and hydration^73^. Extrinsic skin aging is mainly attributed to the repeated exposure to solar UV radiation (photoaging), which causes overproduction of reactive oxygen species (ROS), leading to physical changes in the ECM. ROS are known to induce the expression of proteolytic enzymes, such as matrix metalloproteinases (MMPs), e.g., collagenase; serine proteases, e.g., elastase; as well as the glycosidase enzyme hyaluronidase^74–76^. These are key enzymes in the skin aging process, responsible for the degradation of collagen, elastin, and hyaluronic acid leading to remodelling of ECM and loss of skin elasticity.

To furnish preliminary insights regarding its anti-aging potential, rosemary extract was assessed for its in vitro anti-elastase, anti-collagenase, and anti-hyaluronidase activities. RHE demonstrated good dose-dependent inhibition of elastase activity with IC_50_ value of 57.6 µg/mL close to the reference standard 1,10-phenanthroline (IC_50_ = 25.6 µg/mL). The extract showed mild anti-collagenase and anti-hyaluronidase effects (IC_50_ of 520.2 µg/mL and 448.1 µg/mL, respectively) (Table 4).

**Table 4:** *In-vitro* antiaging potential of *R. officinalis* hexane extract.

Sample	IC_50_ (µg/mL)		
	Elastase	Collagenase	Hyaluronidase
RHE	57.61 ± 2.93	520.2 ± 26.5	448.1 ± 22.8
1,10-Phenanthroline	25.6 ± 1.3	340.8 ± 17.3	234.6 ± 11.9

Interestingly, elastase enzyme has been reported to activate MMP precursors leading to further degradation of ECM^77^. Additionally, elastase was found to degrade decorin, a proteoglycan that binds to and protects collagen fibrils from cleavage by MMP. This renders collagen more susceptible to the proteolytic action of MMP^35^. Inhibition of elastase can hence stop subsequent degradation steps. Pentacyclic triterpenoids, e.g. lupeol and ursolic acid, are known to inhibit elastase^78^. Since collagenase is a zinc-containing enzyme, phenolic compounds, e.g. flavonoids, phenolic acids, phenolic diterpenes, tannins, and tocopherols (known as metal chelators), were reported to inhibit this metalloproteinase^79^.

### In vitro wound healing potential

Wound healing includes the formation and remodelling of new tissues. Migration and proliferation of cells at the wound edge are necessary to close the wound and repair the injured tissue. Many plants have been used in folk medicine to accelerate this process and to prevent infection, such as calendula, for which the wound healing potential was established clinically^80^. In a wound-healing scratch assay, RHE improved the migration and repopulation of keratinocytes at the scratched area and considerably narrowed the scratched gap relative to the control. At 10 µg/mL, RHE showed a closure percentage of 91.85 ± 5.1%, compared to 59.25 ± 3.3 of the control; results superior to calendula hexane and ethanol extracts^81^.

### Cytotoxic activity

Natural anti-aging interventions include the use of medicinal and aromatic plants known to contain bioactive phytochemicals exerting a myriad of pharmacological activities. Foremost is the issue of safety. The chemical composition of certain plant extract can change according to its geographical origin, growing and processing conditions, solvent used as well as the extraction method. Safety assessment of natural products should be extract-specific to better identify their biological prospect. Several rosemary extracts and isolated phytoconstituents demonstrated cytotoxic and anticancer effects against cancer cell lines^22,82–85^. However, little is known about the potential cytotoxic effects of rosemary on normal human cells. RHE revealed very weak cytotoxic effect against normal lung fibroblast WI38 cell line (IC_50_ of 1227.7 ± 14.3 µg/mL) compared with acyclovir (IC_50_ = 48.65 ± 3.2 µg/mL). The extract can be considered relatively safe for use.

### In silico molecular docking study

To rationalize elastase inhibition on a structural level, in silico docking study of the nine major constituents of RHE, as indicated by GCMS analysis, was performed revealing that two components, verbenone and α-amyrin, were able to bind effectively to elastase active site better than the control 1,10-phenanthroline with a Glide G-score of −5.327 and −4.563 kcal/mol, respectively, compared to −4.556 kcal/mol for 1,10-phenanthroline (Table 5). The superior score, especially for verbenone, explains the good inhibitory effect of RHE against elastase enzyme in vitro. Indeed, verbenone was previously reported as potent inhibitor of elastase enzyme with IC_50_ in the picomolar range^86^. The remaining 7 constituents showed docking scores lower than the control and were therefore excluded as being solely responsible for the in vitro anti-elastase activity of RHE.

**Table 5:** Docking scores of RHE major constituents against elastase, hyaluronidase and collagenase as compared to the control, 1,10-phenanthroline.

Compound	Compound structure		Glide G-score		
			Elastase	Collagenase	Hyaluronidase
Verbenone			− 5.327	− 5.281	− 3.829
α-Amyrin			− 4.563	− 3.508	− 3.398
Camphor			− 4.273	− 5.402	− 4.515
β-Amyrin			− 3.515	− 3.765	− 3.339
Lupeol acetate			− 3.267	− 3.465	− 2.072
Octacosane			− 2.424	− 3.903	− 0.698
Triacontane*			*	− 2.95	− 0.885
Doctriacontane*			*	− 4.767	− 0.775
Tetratriacontane*			*	*	*
1,10-Phenanthroline			− 4.556	− 6.931	− 4.714

*Compounds were rejected by the docking engine due to their exceedingly large size.

**Figure 1:**
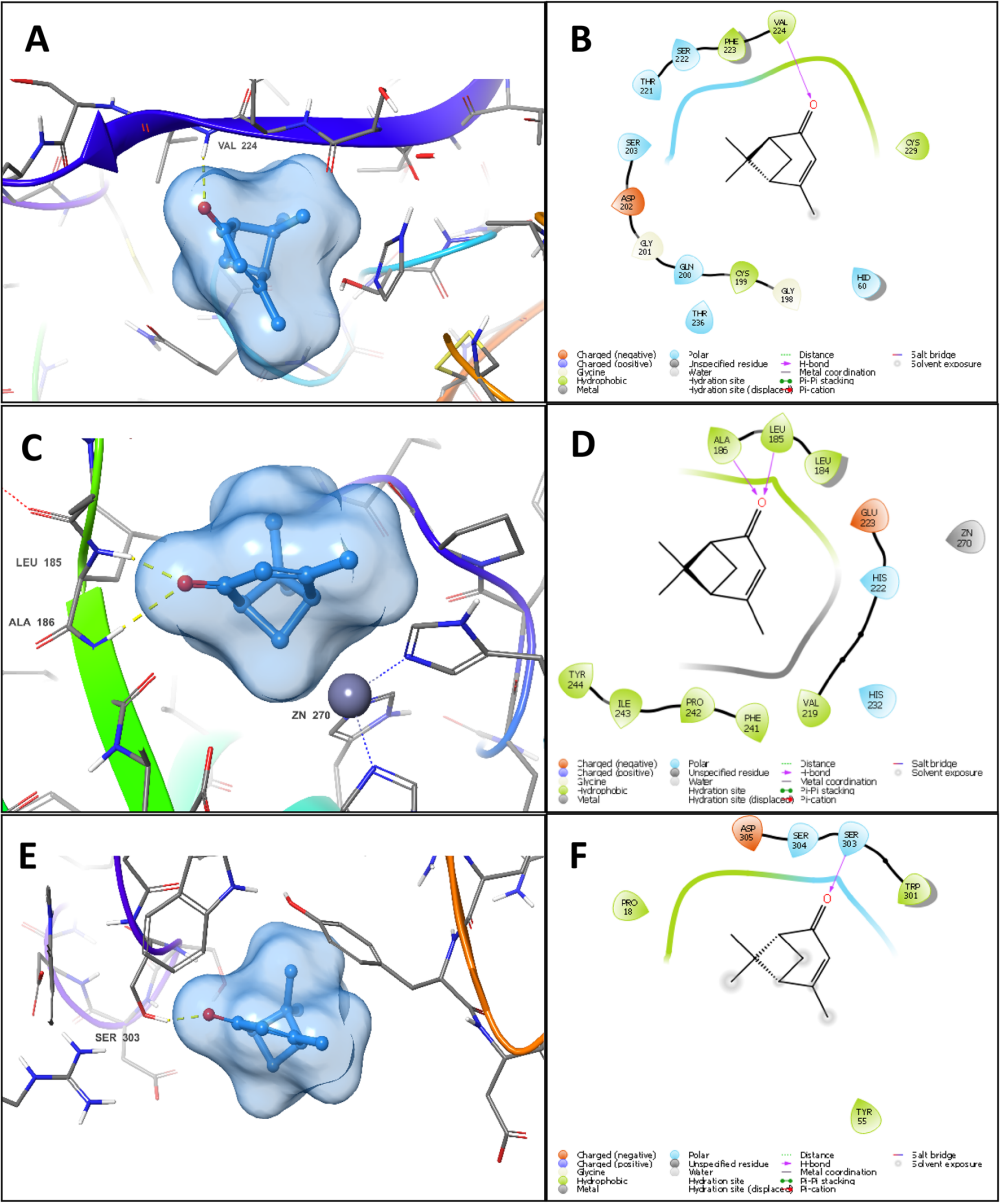
3D docking pose and 2D interaction diagram of verbenone in the binding site of elastase (**A**,**B**), collagenase (**C**,**D**) (showing lack of metal coordination interaction) and hyaluronidase (**E**,**F**) (showing excessive water exposure).

The docking pose of verbenone in elastase revealed the formation of a major hydrogen bond with the backbone amide nitrogen of Val224 through its carbonyl group (Fig. 1A,B). This critical interaction plays a major role in the anchorage of verbenone to the binding site of the enzyme. The rest of the small hydrophobic skeleton then buries itself in the binding site groove forming Van der Waal interactions with the hydrophobic side chains of surrounding residues, with minimal exposure to the aqueous medium. Due to verbenone small size and the presence of a hydrogen bond donor, it can fit easily in elastase active site and form non-covalent interactions that promote enzyme inhibition.

In contrast, the docking poses of verbenone in collagenase and hyaluronidase binding sites are unfavourable. In collagenase, verbenone formed two hydrogen bonds with the backbone nitrogen of Leu185 and Ala186, along with Van der Waal interactions with nearby hydrophobic residues, e.g., Leu185 and Tyr244. However, verbenone failed to interact with the active site zinc, which is crucial for collagenase inhibition. Due to this lack of ligand–metal coordination, verbenone failed to inhibit collagenase enzyme (Fig. 1C,D).

In hyaluronidase binding site, verbenone formed a hydrogen bond to the side chain hydroxyl group of Ser303 (Fig. 1E,F which constitutes an important residue in the binding of hyaluronic acid to hyaluronidase as shown in the co-crystal structure (PDB ID: 1FCV). Furthermore, per-residue interaction scores showed that the hydrogen bond strength of verbenone to Ser303 in hyaluronidase (−0.320 kcal/mol) is greater than hydrogen bond strength of verbenone to Val224 in elastase (−0.286 kcal/mol). However, the overall binding pose of verbenone to hyaluronidase does not qualify it to become an inhibitor due to the superficial binding of verbenone to the active site of hyaluronidase, prohibiting the ligand from immersing itself in the folds of the active site and escaping the aqueous medium. The over-exposure of the hydrophobic skeleton of verbenone to the aqueous medium has made the overall binding of the ligand unstable, and therefore its activity minimal.

**Figure 2:**
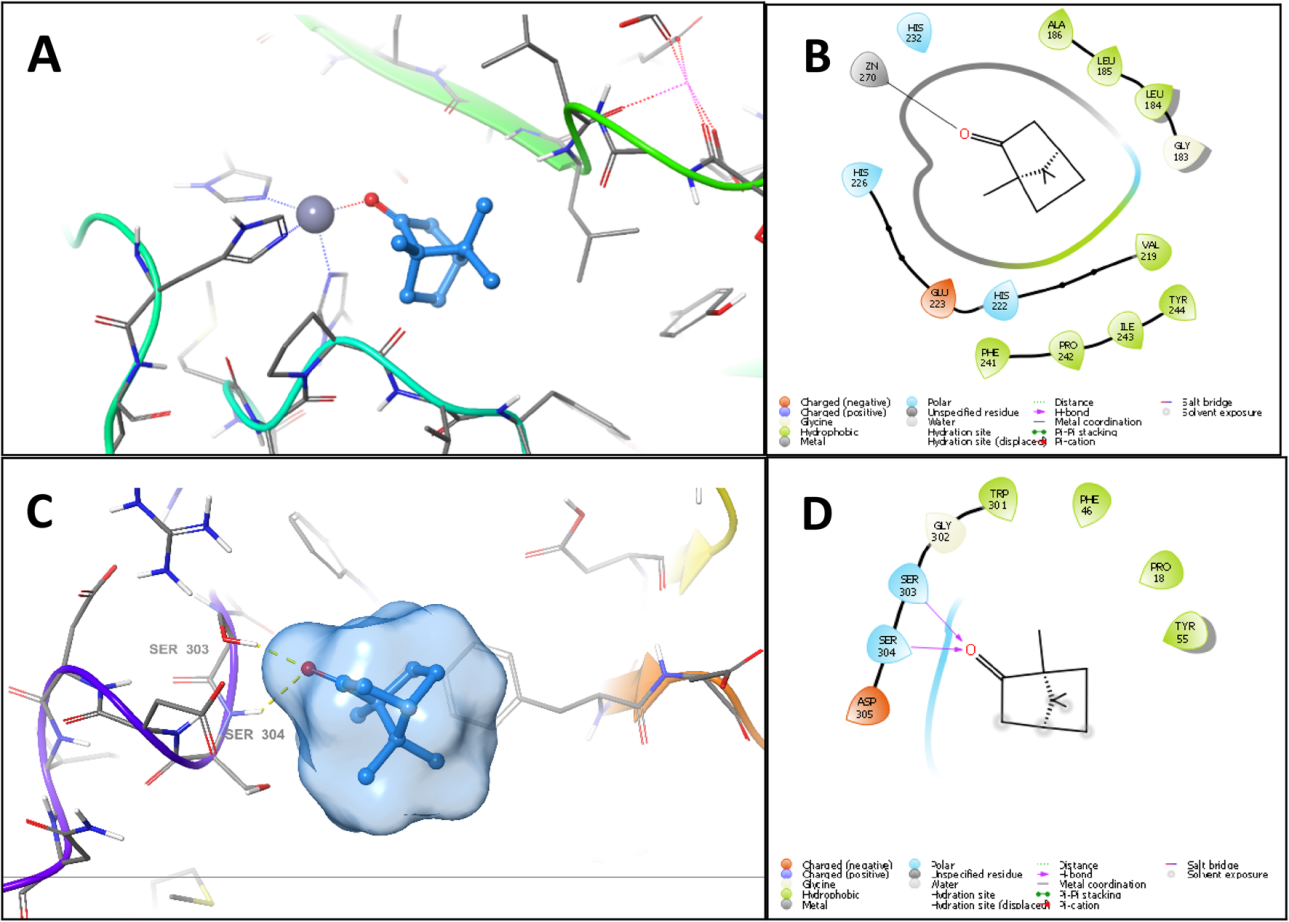
3D docking pose and 2D interaction diagram of camphor in the binding site of collagenase (**A**,**B**) and hyaluronidase (**C**,**D**).

It is noteworthy that in collagenase and hyaluronidase docking experiments, the control 1,10-phenanthroline scored higher than RHE major constituents (Table 5), among which camphor showed the best score and binding pose. The docking experiment of camphor in collagenase binding site showed better binding compared to verbenone. Camphor directed its carbonyl oxygen towards the active site zinc and approached it at 1.98 Å. At this distance, the carbonyl could form a mono-dentate metal chelation interaction with the active site zinc (Fig. 2A,B). Furthermore, the hydrophobic skeleton of camphor was buried among the hydrophobic residues Pro242, Ile243, Leu185, Tyr244, and Tyr214. Despite the good binding pose of camphor in the binding site of collagenase, the expected activity of camphor against collagenase may not be ideal due to the lack of another metal chelation interaction with zinc, since a bi-dentate metal coordination is required for potent collagenase inhibition.

In case of hyaluronidase, camphor formed of hydrogen bonds. The carbonyl oxygen of camphor bonded to the hydroxyl side chain of Ser303 and the backbone amide nitrogen of Ser304 (Fig. 2C,D). Nevertheless, camphor binding to hyaluronidase could not be stable due to the exposure of the hydrophobic skeleton of camphor to the aqueous medium. These findings are in agreement with the in vitro anti-aging results. Verbenone, with its higher docking score and binding mode, may be responsible for the overall anti-elastase activity of the extract. Meanwhile, the docking poses of camphor and verbenone against collagenase and hyaluronidase revealed that these compounds could not inhibit both enzymes, a result that validates our experimental findings.

### Characterization of LNC

The results of the PS distribution and ZP measurements are listed in Table 6. As shown, the PS of blank LNC, 4% RM-LNC (rosemary-loaded lipid nanocapsules), and 10% RM-LNC were 42.28 ± 0.417 nm, 55.20 ± 0.218 nm, and 64.81 ± 1.113 nm, respectively. The obtained PS of blank LNC was comparable to previous results regarding the small size of LNC^87,88^. As observed, rosemary loading into LNC resulted in a significant (*p* < 0.05) increase in PS of RM-LNC, which may be attributed to the increase in the mass of the oily core with subsequent increase in PS^89^. Both blank and rosemary-loaded LNC showed homogenous PS distribution as reflected by the low PDI values (< 0.3). Despite rosemary loading into LNC resulted in significant (*p* < 0.05) increase in PDI values, they still reflect the narrow particle distribution.

**Table 6:** Composition and characterization of blank and rosemary-loaded lipid nanocapsules (RM-LNC).

Formula	Particle size (nm) ± S.D	PDI ± S.D	Zeta potential (mV) ± S.D
Blank LNC	42.28 ± 0.417	0.048 ± 0.001	− 12.6 ± 1.41
Blank LNC gel	45.72 ± 0.079	0.032 ± 0.004	− 11.2 ± 0.70
4% RM-LNC	55.20 ± 0.218	0.175 ± 0.016	− 13.1 ± 0.63
4% RM-LNC gel	56.55 ± 0.384	0.207 ± 0.014	− 13.4 ± 0.91
10% RM-LNC	64.81 ± 1.113	0.262 ± 0.028	− 15.4 ± 2.62
10% RM-LNC gel	66.13 ± 1.306	0.249 ± 0.007	− 15.6 ± 0.89

PDI: polydispersity index, S.D.: standard deviation.

The small PS associated with homogenous distribution is a main characteristic of LNC, which favours its application in topical use. Small PS represents a main advantage due to the increased surface area of the particles forming a dense monolayer and promoting better skin contact, which is needed to achieve high protection against UV radiation^90^. Moreover, previous studies reported the enhanced penetration of the lipid nanocarriers into the epidermal layer as the PS decreases^87,91,92^.

The ZP values of blank LNC, 4% RM-LNC, and 10% RM-LNC were −12.6 ± 1.41 mV, −13.1 ± 0.63 mV, and −15.4 ± 2.62 mV, respectively. The negative charge of the prepared LNC is attributed to the existence of negatively charged phospholipids and the PEG dipoles that took part in the formation of the LNC shell^93–95^. It has been reported that colloidal stability increases as the ZP values increase (≥ ± 30 mV), which is attributed to the electric repulsion between particles. However, the stability of LNC with low ZP values is attributed to steric stabilization of LNC by their tensioactive rigid membrane^33,34^. It can be observed that rosemary-loaded LNC displayed higher negative value of the ZP than blank LNC. A clear explanation was provided by a previous study conducted by Valcourt et al. 2016, which stated that the incorporation of oils into the lipid core of the LNC had a negative impact on the density of PEGylated surfactant at the particle surface with a subsequent increase in the contact area between the lipoid molecules and the external phase and resulted in an increase in the absolute value of the ZP^89^.

### Characteristics of RM-LNC gels

Incorporation of lipid nanocarriers into mucoadhesive gels combined the advantages of a topically delivered formulation with those of nanocarriers in the same final product. These advantages include ease of application, high mucoadhesion with extended skin contact, and slow drug release rate^96,97^.

Based on our previous study, 3% w/w HEC had a viscosity of 30 to 40 poise^98^, which is considered acceptable viscosity for sunscreen gels as reported in previous study^99^. Therefore, 3%w/w HEC was chosen to be added to the LNC dispersion. It was expected that the presence of LNC might have an influence on the measured viscosity of the gel; therefore, the rheological properties of the RM-LNC gels were investigated. In addition, RM-LNC gel was characterized in terms of PS, PDI, and ZP.

Blank LNC, 4%RM-LNC, and 10%RM-LNC gels showed PS equals 45.72 ± 0.079 nm, 56.55 ± 0.384, nm and 66.13 ± 1.306 nm, respectively, where no significant (*p* > 0.05) increase in PS was reported upon LNC incorporation into HEC gel. As observed, all LNC gels are characterized by low PDI values and negatively charged ZP, which indicates their physical stability upon addition of the gelling agent.

The respective viscosity of blank LNC, 4%RM-LNC, and 10%RM-LNC gels were 32.91 ± 1.54, 35.42 ± 3.89, and39.55 ± 2.76 poise, respectively, which are acceptable values for the topical application. The pH values of blank LNC, 4%RM-LNC, and 10%RM-LNC gels were in the range of 6–8, which are considered safe for application as a sunscreen for its photoprotective effect.

### In vivo studies

#### Biochemical analysis

Unprotected exposure to UVB irradiation results in skin damage; this can be assessed by the validation of different biochemical markers. In this study, the suggested protective effect of the prepared RM-LNC in comparison to RHE was investigated by measuring the level of some antioxidant, anti-inflammatory, and anti-wrinkling markers.

##### Antioxidant markers

The non-enzymatic antioxidants, GSH, as well as the antioxidant enzymes, SOD and CAT, were measured in the different studied groups. It is expected that the level of these enzymes decreases in oxidative stress conditions, like exposure to UV irradiation. As listed in Table 7, the marked decrease in their level in case of the positive control group compared to the negative one (*p* < 0.05) was abolished in the groups administered with the RHE as well as the RM-LNC gels, suggesting that rosemary can replenish antioxidants. This validates the initial in vitro screening, sheds light on skin penetration of applied formulae, and confirms their effectiveness in biological systems. The more potent effect of RM-LNC gels compared to the RHE (*p* < 0.05) is expected based on the above discussed in vitro study as well as the visual examination of the dorsal rats’ skin before sacrificing. The enhanced RHE solubilization and release, reduced PS, as well as the elastic properties of the designed LNC can assure an enhanced skin penetration and photoprotective effect. Some previous studies have reported that the PS range recorded for prepared formulae facilitates drug penetration and accumulation into the skin, which allows a localized and site-specific drug effect^87,100^.

**Table 7:** Effect of UVB-irradiation and different formulations on the oxidative stress, inflammatory and wrinkling markers in rats.

Group	Antioxidant parameters			Anti-inflammatory parameters			Anti-wrinkling parameters			
	Catalase	SOD	GSH	IL—6	IL—1 beta	NF—KB	GM—CSF	MMP1	Elastase	Neprilysin
	U/g tissue	U/g tissue	Pg/g tissue	Pg/g tissue	Pg/g tissue	ng/g tissue	Pg/g tissue	ng/g tissue	ng/g tissue	Pg/g issue
NC	38.7 ± 1.14	45.6 ± 1.29	50.6 ± 1.10	14.1 ± 1.18	20.4 ± 1.2	23.4 ± 1.19	6.4 ± 0.87	2.1 ± 0.68	1.6 ± 0.85	26.7 ± 1.68
PC	7.8 ± 0.95*	12.3 ± 1.54*	17.4 ± 1.13*	40.1 ± 1.85*	46.1 ± 0.84*	55.2 ± 0.75*	29.1 ± 1.25*	9.5 ± 1.06*	7.9 ± 1.05*	51.2 ± 1.15*
T1	14.3 ± 0.92*^@^	19.7 ± 0.65*^@^	24.5 ± 1.22*^@^	27.6 ± 1.65*^@^	19.1 ± 1.35*^@^	36.5 ± 1.16*^@^	19.1 ± 1.35*^@^	6.7 ± 0.85*^@^	5.6 ± 0.79*^@^	40.1 ± 1.12*^@^
T2	29.5 ± 1.35*^@#^	24.8 ± 0.98*^@#^	29.5 ± 1.35*^@#^	23.4 ± 1.42*^@#^	29.7 ± 1.3*^@#^	32.4 ± 1.18*^@#^	15.7 ± 1.2*^@#^	5.4 ± 0.98*^@#^	4.6 ± 1.02*^@#^	35.2 ± 1.4*^@#^
T3	38.2 ± 1.64*^@#a^	32.9 ± 0.88*^@# a^	38.2 ± 1.64*^@# a^	16.9 ± 1.85*^@# a^	23.1 ± 1.06*^@# a^	25.4 ± 1.06*^@# a^	9.2 ± 1.05*^@# a^	3.2 ± 0.56*^@# a^	2.3 ± 0.65*^@# a^	28.9 ± 1.8*^@# a^
T4	12.3 ± 0.65*^@#+++^	15.8 ± 1.05*^@#+++^	20.4 ± 1.16*^@#+++^	32.1 ± 1.88*^@#+++^	23.7 ± 1.23*^@#+++^	40.3 ± 1.06*^@#+++^	23.7 ± 1.23*^@#+++^	7.7 ± 0.62*^@#+++^	6.4 ± 0.56*^@#+++^	44.6 ± 1.65*^@#+++^

NC: Negative control (normal rats), PC: positive control (subjected to UVB irradiation and received no treatment), while T1, T2, T3 and T4 received RHE, 4%-RM-LNC gel, 10% RM-LNC gel and plain LNC gel. Each value is presented as mean ± standard error of the mean (SE) for 10 rats.

*Statistically significantly different from the normal control group (P < 0.05).

^@^Statistically significantly different from the positive control group (P < 0.05).

^#^Statistically significantly different from the T1 group (P < 0.05).

^a^Statistically significantly different from the T2 group (P < 0.05).

^+++^Statistically significantly different from the T3 group (P < 0.05).

##### Anti-inflammatory activity

UVB exposure up-regulates inflammatory cytokines causing skin damage^101^. The values seen in Table 7 show that UVB exposure induced a significant increase in the inflammatory markers (IL-1β, IL-6, and NF-kB) of the positive control group compared to those of the negative control (*p* < 0.05). This effect decreased in case of the groups pre-treated with RHE and RM-LNC (*p* < 0.05). However, the effect of the RM-LNC formulae was higher than the RHE (*p* < 0.05). As discussed above, the superior effect of the RM-LNC against the inflammatory reactions induced by UVB irradiation can be due to the intrinsic properties of the designed LNC, which can breach the skin barrier and penetrate deeply into the inner skin layers. This reduction in photo-inflammation was evidenced by decreased erythema, edema, and skin thickness. The observed anti-inflammatory effect can be attributed to the rich triterpenoids and phenolic diterpenes content of rosemary. The major components of RHE, α- and β-amyrin, were reported to exert anti-inflammatory activity through the activation of the cannabinoid receptors^102^. Carnosic acid, carnosol and rosmanol suppress nitric oxide and TNF-α, downregulate COX2 expression and inhibit PGE2 synthase-1, iNOS and 5-lipoxygenase^103–105^. Moreover, the anti-inflammatory activity of the monoterpenoid eucalyptol was well established through the suppression of lipopolysaccharide-induced pro-inflammatory cytokines, e.g. IL-1β, IL-6, and NF-kB^21^.In addition, the synergism with other existing phytoconstituents cannot be precluded.

##### Anti-wrinkling markers

Exposure to UVB irradiation triggers the production of free radicals, which upregulate the production of matrix metalloproteinases (MMPs). Degradation of the collagen and elastin network is caused by MMPs^106^, leading to skin wrinkling. UVB irradiation causes keratinocytes to secrete IL-1, which stimulates GM-CSF secretion and triggers fibroblasts to stimulate their expression of neprilysin/NEP, which results in the deterioration of the three-dimensional fibre networks and the loss of skin elasticity and wrinkles formation^107,108^. As observed in Table 7, the levels of MMP1, GM-CSF, neprilysin, and elastase are higher in the positive control group than in the negative control one (*p* < 0.05). Application of RHE or RM-LNC protected the skin from wrinkling and aging (*p* < 0.05). As expected, the photoprotective effect of the studied formulae was significantly superior (*p* < 0.05). Surprisingly, the major ingredient in RHE, α-amyrin, did not provide in vitro protection against UVB damage in earlier reports^43^, suggesting that the observed photoprotection can be mediated by potentiating interactions among several constituents, including the minor ones.

#### Cutaneous irritancy test

Skin applied formulae are required to be innocuous, neither creating irritancy nor allergenicity^109^. Both the plain and rosemary-loaded LNC gels were tested to evaluate the safety of each component of the preparations. The results shown in Table 8 proved the non-irritancy of tested gels (PII < 2) all over the period of the experiment (72 h). Statistical analysis shows that the formalin solution (group 2) was significantly irritant (*p* < 0.001) compared to the control group (group 1) and all the gels, whereas there was no significant difference (*p* > 0.05) between all gels and the control group.

**Table 8:** Cutaneous irritancy test.

Rat	Negative control		Positive control		T1		T2		T3		T4	
	Er	Ed	Er	Ed	Er	Ed	Er	Ed	Er	Ed	Er	Ed
1	1	0	4	3	1	0	1	0	2	0	1	0
2	0	0	3	4	1	0	1	0	1	1	2	1
3	0	0	4	4	1	0	0	0	2	1	2	1
4	1	0	4	3	2	1	0	1	2	0	2	1
5	1	0	4	4	2	1	1	0	1	1	2	1
6	1	0	4	4	1	1	1	0	1	1	1	0
Average	0.66	0	3.83	3.66	1.33	0.5	0.66	0.166	1.5	0.66	1.66	0.66
PII	0.66	± 0.15	7.5^+++^	± 0.16	1.83^†a^	± 0.12	0.83^†a^	± 0.13	2.16^†a^	± 0.14	2.33^†a^	± 0.16

^+++^Significant (*p* < 0.001) when compared to group 1.

^a^Significant (*p* < 0.001) when compared to group 2.

^†^Non-significant (*p* > 0.05) when compared to group 1.

#### Histopathological examination

**Figure 3:**
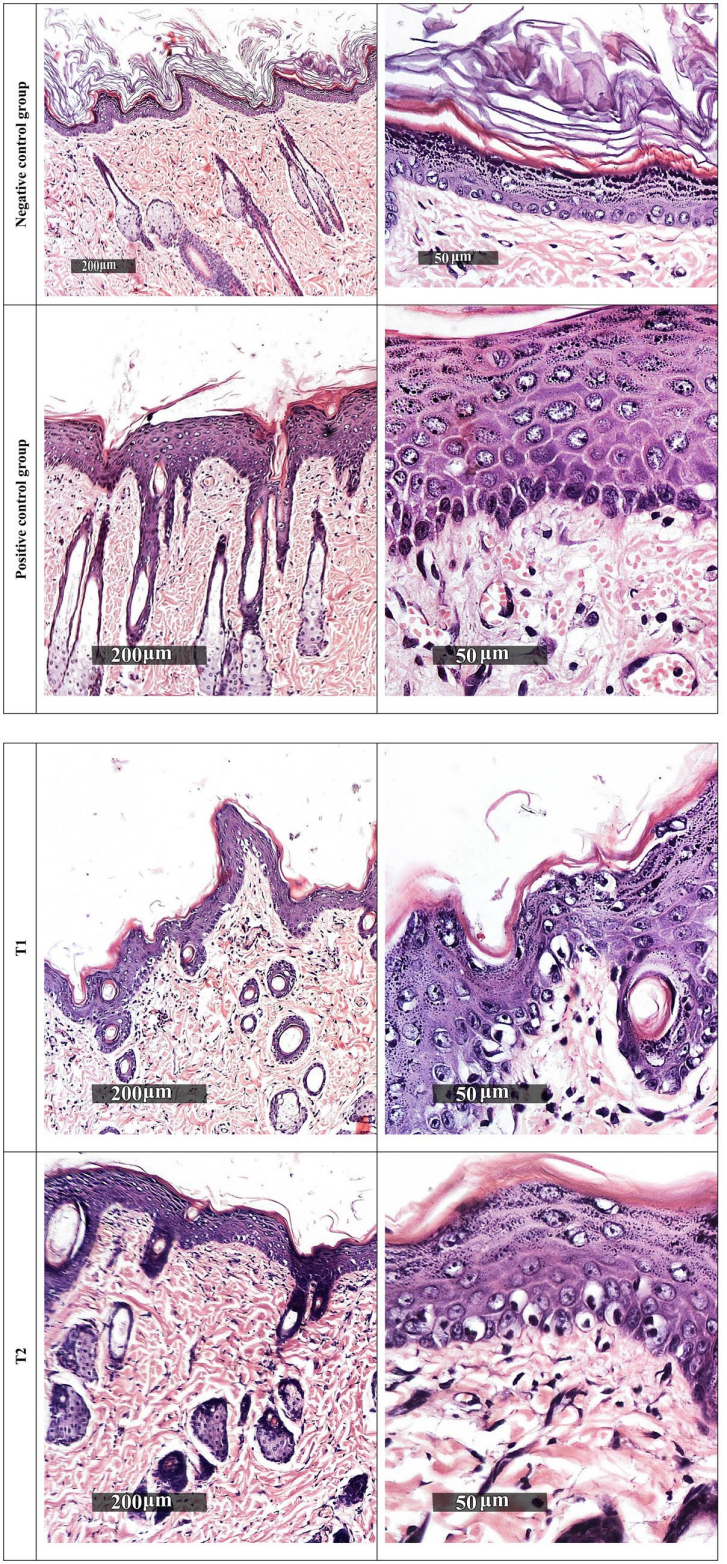

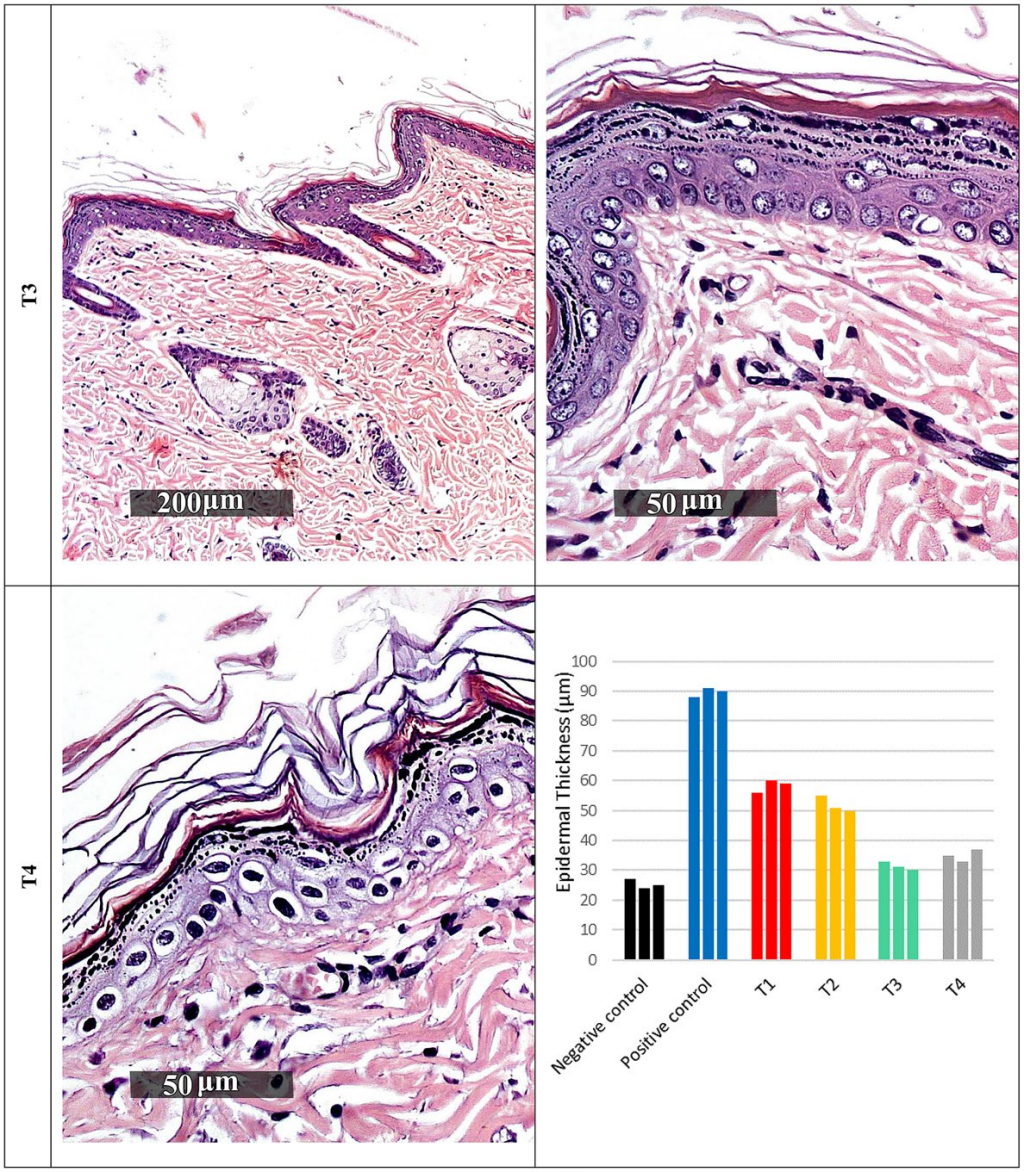
Histopathological examination of rat dorsal skin. Negative control: normal rats, positive control: subjected to UVB irradiation and received no treatment, while T1, T2, T3 and T4 received RHE, 4%-RM-LNC gel, 10% RM-LNC gel and plain LNC gel.

The photos of the skin subjected to histopathological study are displayed in Fig. 3. The negative control group demonstrated normal morphological features of skin layers including thin intact epidermal layer with well-organized apparent intact subcellular details of different keratinocytes in different zones, intact dermal layer with abundant collagen fibres, minimal inflammatory cells infiltrates, and normal vasculatures. On the other hand, the positive control group revealed significant increase of epidermal thickness with alternated areas of apparent intact or pyknotic basal cells layer accompanied with mild to moderate dermal mononuclear inflammatory cells infiltrates as well as congested subepidermal blood vessels and focal hemorrhagic zones. A certain improvement of the condition can be observed in rats treated with RHE; however, samples revealed moderate reduction of epidermal thickening with persistence of degenerative changes records of basal cell layer, mild subepidermal mononuclear cells infiltrates, and congested blood vessels.

Group T2 samples showed moderate reduction of epidermal thickening as shown in Fig. 3, with persistence of degenerative changes records of basal cell layer and mild subepidermal mononuclear cells infiltrates. However, normal subepidermal vasculatures were recorded.

Group T3 samples demonstrated almost intact well-organized skin layers with minimal records of abnormal morphological features all over epidermal and dermal layers.

Finally, group T4 showed abundant records of degenerated and pyknotic epidermal keratinocytes with normal epidermal thickness ranges accompanied with mild occasional sub-epidermal inflammatory cells infiltrates as well as congested BVs.

Previous studies have established the anti-inflammatory and photoprotective activities of rosemary polyphenols and polar extracts^19,110^. However, this is the first report of the photoprotective potential of rosemary hexane extract which is often regarded as agro-industrial processing waste during the extraction of polyphenols.

## Materials and methods

### Materials

Kolliphor® HS 15 (Solutol® HS 15), a mixture of free polyethylene glycol 660 and polyethylene glycol 660 hydroxy stearate) was purchased from Sigma-Aldrich, Zwijndrecht, Netherlands. Labrafac lipophile WL1394 (triglyceride medium chain, caprylic/capric TG) and soybean phosphatidylcholine (Epikuron™170 (EP) were a kind gift from Gattefosse, Saint-Priest, France. Sodium chloride, methanol, and n-hexane were purchased from Adwic, El-Nasr Pharmaceutical Co., Cairo, Egypt. Hydroxyethyl cellulose (HEC) (2% solution has a viscosity of 640.9 cP) was kindly supplied by Memphis Co., Cairo, Egypt.

### Plant material and extraction

Leaves from three nearby shrubs of *Rosmarinus officinalis* L. were collected at the Medicinal Plants Station, Faculty of Pharmacy, Ain Shams University (Cairo) in July 2019 and were kindly authenticated by Mrs. Therese Labib, plant taxonomist at the Ministry of Agriculture. Experimental research on plant, including the collection of plant material, complied with relevant institutional, national, and international guidelines and legislation. The study did not include any species at risk of extinction or endangered species. A voucher specimen was deposited at the herbarium of Pharmacognosy Department, Faculty of Pharmacy, Ain Shams University (Cairo) for referencing (PHG-P-RO-328). Fresh leaves were ground in a kitchen-type milling machine and macerated in distilled n-hexane (2 × 3 L) for 48 h. Extraction was assisted by sonication for three intervals 10 min each. After filtration, the extract was evaporated under reduced pressure at 40 °C using a rotary-type evaporator (Büchi, Switzerland) to yield a green greasy solid residue (2.1% w/w) which was stored at − 20 °C until needed.

### GC-MS analysis

A Shimadzu GC-MS-QP2010 was used to perform the GC-MS analyses (Shimadzu Corporation, Kyoto, Japan) as recently reported^111^.

### LC-ESI-HRMS analysis

For the chromatographic separation, 6530 Q-TOF LC/MS (Agilent Technologies) equipped with an autosampler (G7129A), a Quaternary Pump (G7104C), and a Column Compartment (G7116A) was used. The injection volume was 5 μL. The analytes were separated on a Zorbax RP-18 column (150 mm × 3 mm, 2.7 μm) in a flow rate of 0.3 mL/min. The mobile phase was composed of solvent A (aqueous formic acid, 0.1% v/v) and solvent B (0.1% formic acid/acetonitile). A gradient mode was implemented as follows; at t = 0–2 min, solvent A/solvent B (90/10); at t = 10 min, solvent A/solvent B, (80/20); at t = 52–80 min, 100% solvent B. Mass spectra were acquired using ESI in negative ionization mode with a capillary voltage of 4500 V. The mass spectra were recorded in the *m/z* range of 50–3000 m/z. The gas temperature and drying gas flow rate were 200 °C and 8 L/min, respectively. The skimmer and fragmentation voltages were set at 65 and 130 V, respectively, and collision energy was 10 V. The nebulization pressure was 58 psi. Data processing was performed using MassHunter workstation B.06.00 (Agilent Technologies, 2012) and compounds were tentatively identified according to their mass spectra, accurate mass and retention time, in comparison with literature.

### In vitro antioxidant activity

#### DPPH radical scavenging activity

The scavenging activity of the stable 2,2-diphenyl-1-picrylhydrazyl (DPPH) free radical by RHE was assessed according to the method reported by Boly et al.^112^.

#### ABTS radical scavenging assay

This assay was carried out according to the method previously reported by Arnao et al.^113^.

#### FRAP assay

The method is based on the reduction of a ferric-tripyridyltriazine (Fe^3+^-TPTZ) complex to its intensely blue ferrous form, at low pH, as previously described^114^.

### In vitro anti-aging potential

#### Anti-elastase assay

The elastase inhibitory activity was assessed fluorimetrically using the EnzCheck® elastase assay kit (Molecular Probes, Laiden, Netherlands). A 1 mg/mL stock solution of the substrate (DQ elastin) was prepared in deionized water. Porcine pancreatic elastase stock solution was prepared in deionized water at 100 U/mL and dilutions were made in Tris–HCL buffer. Pre-incubation of 50 µL of different dilutions of the extract (inhibitor) with 100 µL of the enzyme was done for 15 min followed by the addition of the substrate (50 µL). Controls were prepared with buffer instead of extract. Fluorescence intensity was continuously measured for 20 min in a fluorescence microplate reader equipped with standard fluorescein filters (λ_ex_ 505 nm, λ_em_ 515 nm). Subtraction of background fluorescence was done in no-enzyme wells. 1,10-Phenathrolinewas used as a standard elastase inhibitor. The IC_50_ is the concentration of the extract required to inhibit 50% of elastase activity.

$${\text{The percentage of elastase inhibition }}(\%) = (1-S/C)\times100$$

where S is the corrected fluorescence of the extract samples and C is the corrected fluorescence of the control.

#### Anti-collagenase assay

The evaluation of collagenase inhibitory activity was performed fluorimetrically in a microplate reader. Briefly, the self-quenched BODIPY conjugate of gelatin (Type B) was used as a fluorogenic substrate to monitor the activity of collagenase (Biovision, CA, USA). Collagenase stock solution was prepared in 50 mM Tricine buffer at 0.8 U/mL and the substrate (BODIPY) was dissolved in Tricine buffer to 2 mM. One microliter of different concentrations of the extract (1, 10, 100, and 1000 µg/mL) was incubated with the 5 µL collagenase in buffer and 44 µL Tricine buffer for 15 min before adding the substrate. (1, 10)-Phenanthroline was used as a positive control. The reaction is initiated by mixing two µL of the substrate with the previous reaction mixture. Negative controls were prepared with the buffer. Fluorescence intensity was monitored (λ_ex_ 490 nm, λ_em_ 520 nm, 515 nm cut-off) in a kinetic mode at 37 °C for 30–60 min.

$$\text{The percentage of collagenase inhibition }(\%) = (1-S/C)\times100$$

where S is the corrected fluorescence of the extract sample and C is the corrected fluorescence of the control.

#### Anti-hyaluronidase assay

A turbidimetric assay was performed to assess the hyaluronidase inhibitory activity using QuantiChrom™ Hyaluronidase Inhibitor Screening Assay Kit (BioAssay Systems, CA, USA). Bovine hyaluronidase (type-1-S, Sigma Aldrich, St. Louis, MO, USA) was diluted to 10 U/mL in buffer. From this solution, 40 µL were transferred to a 96-well plate. Instead, enzyme buffer (40 µL) was used for No Enzyme Control (NEC), while hyaluronidase (40 µL) was used for No Inhibitor Control (NIC). To the NIC and NEC wells, 20 μL DMSO were added and to the sample wells 20 µL of the desired extract concentrations were added followed by incubation for 15 min at room temperature. The substrate (40 µL) was added and the plate was incubated for 20 min. The stop reagent (160 µL) was used to halt the enzymatic reaction and forms turbidity with any residual hyaluronic acid. Plate was incubated for 10 min and the optical density was read at 600 nm. The percentage inhibition was calculated as follows:

$$\% \text{ Inhibition}=1-[(OD_{NEC}-OD s_{ample})/(OD_{NEC}-OD_{NIC})]\times100$$

where OD_NEC_, OD_NIC_, and OD_sample_ represent the optical density values of the No Enzyme Control, No Inhibitor Control, and the extract.

#### Scratch-wound healing assay

Effect of RHE on keratinocytes migration was evaluated using the scratch assay as previously described^115^.

#### Cytotoxicity assay

The cytotoxicity of RHE extract was evaluated against human normal lung fibroblasts cell line WI-38 using MTT (3-(4,5-dimethylthiazol-2-yl)2,5-diphenyl tetrazolium bromide) assay^116^ as recently described^111^.

### In silico molecular modelling

All docking experiments were done using Glide docking engine (Schrödinger Release 2020–4: Glide, Schrödinger, LLC, NY, 2020). The crystal structures of the three target enzymes, elastase (PDB ID: 1ELC, 1.75 Å), collagenase (PDB ID: 2D1N, 2.37 Å), and hyaluronidase (PDB ID: 1FCV, 2.65 Å), were downloaded from the Protein Data Bank (PDB). The crystal structures were co-crystallized with non-covalent inhibitors. The PDB files were imported into Maestro and prepared using the protein preparation wizard and standard protein preparation protocol. The docking grid was then generated using the grid receptor grid generation module and the co-crystallized ligand was selected as the grid centre. The ligands were then imported and prepared using Ligprep module and standard ligand preparation protocol. Molecular docking was carried out using Glide Standard Precision and no constraints. Per-residue interaction scores were calculated for selected ligands and residues during docking re-runs using the same procedures.

### Preparation of blank and rosemary-loaded LNC

Blank LNC were prepared using the phase inversion method with three temperature cycles^87^. In brief, aqueous phase composed of Solutol® HS 15 (1 g), sodium chloride (0.1 g), and demineralized water (3 g) was mixed with the oily phase of Labrafac (0.9 g) and EP (0.1 g) in a closed container under magnetic stirring for 10 min. The mixture was heated up to 85 °C under magnetic stirring, followed by cooling to 55 °C to ensure phase inversion from w/o emulsion to o/w emulsion. The heating/cooling cycle was repeated two times followed by the addition of 5 ml of cold water (0–2 °C) with magnetic stirring. The LNC dispersions obtained were kept at 4 °C for further investigation. Rosemary-loaded LNC (RM-LNC) were prepared using the same procedure, where the rosemary extract (4%w/w or 10%w/w) was dissolved in the oily phase by magnetic stirring before mixing with the aqueous phase, then the procedure was completed as previous.

### Particle size distribution and zeta potential measurements of the prepared LNC

The particle size (PS), polydispersity index (PDI), and zeta potential (ZP) for the prepared blank and RM-LNC were determined at 25 °C using a laser diffraction particle size detector (Zetasizer; Malvern Instruments, Malvern, UK) after suitable dilution.

### Preparation and characterization of blank and RM-LNC-based gels

Gels based on LNC were prepared by gradual sprinkling of HEC as gelling agent into the LNC dispersions under magnetic stirring until complete hydration. The gel was sonicated for dissipation of entrapped air and stored at 4 °C for further evaluation^117^.

The PS, PDI, and ZP of blank and RM-LNC-based gels were measured as previously described after suitable dilution with deionized water with magnetic stirring. Viscosity measurements were conducted using viscometer (Brookfield Engineering Laboratories Inc., Model HADV-II, USA) connected to a digital thermostatically controlled circulating water bath (Polyscience, Model 9101, USA) with spindle 52 at a speed of 50 rpm 25 ± 0.1 °C. Equilibration of the sample for 5 min was made following loading of the viscometer. All studies were performed in triplicates and the average was taken^118^. The pH of 5% w/w dispersions of the gels in water was determined using pH meter.

### In vivo study

#### Animals

The experiment was performed on the hairless skin of adult male Wistar rats weighing between 180 and 220 g (6–8 weeks old) obtained from the animal house of the National Research Center, Cairo, Egypt. The animals were housed in plastic cages and kept in a conditioned atmosphere at 22 ± 3 °C and humidity 50–55% with 12 h light/dark cycles. They were fed standard pellet chow (El-Nasr chemical company, Cairo, Egypt) and were permitted free access to water. This study was conducted in accordance with ethical procedures and policies approved by the Institutional Animal Care and Use Committee of Cairo University, Egypt (Ethical Approval Number IACUC-CU-III-F-42–20). The study followed the recommendations in the ARRIVE guidelines.

### Experimental design

The dorsal side of the rats was shaved 24 h before the beginning of the experiment. Sixty rats were randomly divided into six groups, each containing 10 animals: a negative control group (C1) was not exposed to irradiation; a positive control group was subjected daily to UVB irradiation for 10 consecutive days and received no treatment. The other four groups named T1, T2, T3, and T4 received RHE, 4%-RM-LNC gel, 10% RM-LNC gel, and plain LNC gel, respectively, daily one hour before the UVB exposure A UV lighter (peak emission was 302 nm, CL-1000 M, UVP, Upland, CA, USA) was used for UVB irradiation. UVB irradiation doses were 40–80 mJ/cm^2^ (exposure time was 15–30 s) and the lamp was fixed 5 cm above the platform where rats were placed^119^.

### Tissue preparation

At the end of the experiment, rats were anesthetized by ketamine (85 mg/kg, i.p.), euthanized by cervical dislocation, and the treated skin of each rat was dissected out into two halves. The first half of the dorsal skin of rats was preserved in 10% formalin for histopathological examination. The other half of skin samples were homogenized and subjected to biochemical estimation of the antioxidant, anti-inflammatory, and anti-aging activities of the prepared RM-LNC gels in comparison with the RHE.

### Biochemical analysis

#### Antioxidant activity

The level of catalase (CAT), reduced glutathione (GSH), and superoxide dismutase (SOD) reactive substances was estimated as reported previously^120,121^ in the homogenate. Catalase (CAT) ELISA Kit was purchased from Hubei, China, Rat Superoxide Dismutase (SOD) ELISA Kit was purchased from MyBioSource, San Diego, US, and Glutathione peroxidase (GSH) was purchased from ShangHai BlueGene Biotech CO., China.

#### Anti-inflammatory activity

The level of interleukin 1 beta (IL-1β), interleukin 6 (IL- 6), and nuclear factor-kappa B (NF-κB) was determined using an enzyme-linked immunosorbent assay (ELISA) kit. Rat NF-κB ELISA Kit and Interleukin 6 (IL-6) were purchased from CUSABIO, Inc., Wuhan, Hubei, China. Rat interleukin 1 beta (IL-1β), ELISA Kit was purchased from MyBioSource, San Diego, US.

#### Anti-wrinkling and anti-photoaging activity

The level of matrix metalloproteinase (MMP-1), granulocyte–macrophage colony-stimulating factor (GM-CSF), neprilysin, and elastase enzymes were determined using an enzyme-linked immunosorbent assay (ELISA) kit. Rat Granulocyte–Macrophage Colony Stimulating Factor (GM-CSF), Rat Elastase ELISA Kit, and Rat Neprilysin ELISA Kit were purchased from MyBioSource, San Diego, US. Matrix metalloproteinase (MMP-1) ELISA kit was purchased from Lifespan Bioscience, North America.

### Histopathological study

Skin specimens obtained from the rats’ dorsal skin were fixed and embedded in paraffin for histopathological studies. Paraffin bees wax tissue blocks were prepared for sectioning at a thickness of four µm by sledge microtome. The obtained tissue sections were collected on glass slides, de-paraffinized, and then stained by hematoxylin and eosin stain for examination using the light electric microscope (Optika B 150, Optika Microscopes, Italy).

### Cutaneous irritation

The irritancy of the RM-LNC gels was evaluated according to the method previously described^122^. The dorsal side of the rats was shaved 24 h before the beginning of the experiment. The animals were divided into 6 groups each containing 6 rats: Group 1 served as control (no treatment), group 2 received 0.8% v/v aqueous formalin solution as a standard irritant^123^, groups T1, T2, T3, and T4 received RHE, 4%-RM-LNC gel, 10% RM-LNC gel, and plain LNC gel. An amount of 100 mg gel (or formalin solution) was applied once daily for 72 h. The application sites were examined for edema and erythema at 24 and 72 h and graded (0–4), as shown in Table 9, according to a visual standard score^124^; the final score represents the average of the 24 and 72 h readings. The primary irritancy index (PII) was determined for each preparation by adding the edema and erythema scores; the formulations were accordingly classified as non-irritant if PII < 2, irritant if PII = 2–5, and highly irritant if PII = 5–8.

**Table 9:** Evaluation of skin reactions.

Skin reaction	Score
**Erythema formation (Er.)**	
None	0
Very slight erythema	1
Well defined erythema	2
Moderate to severe erythema	3
Severe erythema and scar formation	4
**Edema formation (Ed.)**	
None	0
Very slight edema	1
Slight edema (edges of area well defined by definite raising)	2
Moderate edema (area raised approximately 1 mm.)	3
Severe edema (raised more than 1 mm. and extending beyond area of exposure	4

### Statistical analysis

The *invitro* data were compared using one-way analysis of variance, followed by multiple comparisons of Tukey–Kramer test using Graph Pad Instat® software (GraphPad4 Software, La Jolla, CA). The significance level was at p < 0.05. Data obtained from in vivo study were expressed as the mean of three experiments ± the standard deviation (SD) or ± the standard error of mean (SEM) and were analysed using one-way analysis of variance (ANOVA), followed by the least significant difference procedure using SPSS® software (SPSS, Inc., Chicago,Illinois, USA). Statistical differences yielding p < 0.05 were considered significant.

## Conclusions

Bioactive natural products and plant extracts inspired by traditional medicine are increasingly expanding the anti-aging and photoprotective therapeutic arsenal especially with the increasing life expectancy and the green shift towards the use of natural health care products. With its content of phenolic diterpenes, triterpenoids, monoterpenoids, and long chain hydrocarbons, rosemary hexane extract demonstrated interesting in vitro anti-elastase, antioxidant, and wound healing properties associated with no cytotoxicity, representing a cost-effective and relatively safe anti-aging approach*. *In silico molecular modelling posed verbenone as the main constituent responsible for the anti-elastase activity of the extract through its significantly high docking score and favourable binding mode. The findings were further consolidated with in vivo results where *Rosmarinus officinalis* hexane extract, formulated in lipid nanocapsules-based mucoadhesive gel, provided UV-protection, restored the antioxidant biochemical state, decreased the level of inflammatory and wrinkling markers, and improved epidermal and dermal histological features in UV-irradiated rat model. The feasibility of synergy with known antioxidant and photoprotective natural products and the use of systemic photoprotection in conjunction with topical routes are yet to be explored.

## Data Availability

The data supporting this study are available upon request to Nehal Ibrahim (nehal.sabry@pharma.asu.edu.eg).
